# Development of a human colorectal carcinoma cell-based platform for studying inducible nitric oxide synthase expression and nitric oxide signaling dynamics

**DOI:** 10.3389/fmolb.2025.1637230

**Published:** 2025-07-17

**Authors:** Xi Chen, Elizabeth A. Grimm, Yong Qin

**Affiliations:** ^1^ Department of Pharmaceutical Sciences, School of Pharmacy, The University of Texas at El Paso, El Paso, TX, United States; ^2^ Department of Melanoma Medical Oncology, MD Anderson Cancer Center the University of Texas, Houston, TX, United States

**Keywords:** iNOS, gene expression, nitric oxide, colorectal adenocarcinoma cells, nitrosative stress

## Abstract

**Introduction:**

Inducible nitric oxide synthase (iNOS) plays a critical role in inflammatory signaling and tumor immunology, contributing to both pro- and anti-tumor effects depending on the cellular context. While iNOS induction has been linked to immune activation and tumor progression, its expression in cancer cells is highly variable and often inconsistently reported across different tumor models. To address this gap, we developed a well-defined *in vitro* platform using the human colorectal adenocarcinoma cell line DLD-1 to model stimulus-dependent iNOS expression and nitric oxide (NO) signaling.

**Methods:**

DLD-1 cells were stimulated with a pro-inflammatory cytokine cocktail (lipopolysaccharide [LPS], interleukin-1β [IL-1β], and interferon-γ [IFN-γ]), resulting in marked upregulation of iNOS at both the mRNA and protein levels. iNOS specificity was confirmed using targeted siRNA knockdown. Functional assessment of NO production was performed using the Nitrate/Nitrite Colorimetric Assay Kit and the ENO-30 NOx Analyzer. Induction of iNOS was further associated with elevated levels of reactive nitrogen species (RNS), reactive oxygen species (ROS), and protein nitration, including 3-nitrotyrosine, detected by immunohistochemistry and Western blot.

**Results:**

Upon stimulation, DLD-1 cells consistently expressed enzymatically active, full-length human iNOS and produced biologically relevant levels of NO and downstream nitrosative stress markers. Treatment with selective iNOS inhibitors significantly reduced nitrite accumulation, confirming the functional activity of iNOS and the model’s applicability for pharmacologic evaluation of NO-modulatory compounds.

**Discussion:**

Our findings establish the DLD-1 cell line as a reproducible and well-controlled in vitro system for studying inducible iNOS expression and downstream NO/RNS signaling in human epithelial cancer cells. This platform provides a valuable tool for mechanistic studies, screening of iNOS-targeted agents, and resolving discrepancies in iNOS detection across experimental models in cancer biology.

## 1 Introduction

NO is a multifunctional signaling molecule involved in diverse physiological and pathological processes, including vasodilation, neurotransmission, immune modulation, and tumor progression (Kim and Thomas, 2022; Ramírez-Patiño et al., 2022; Sahebnasagh et al., 2022). The role of NO in cancer is particularly complex and concentration-dependent. While low concentrations of NO (in the pM to nM range) can promote tumor survival and immune evasion, higher concentrations (in the µM range) are associated with cytotoxicity and tumor suppression (Kim and Thomas, 2022; Ramírez-Patiño et al., 2022). This biphasic effect contributes to the contradictory reports in the literature regarding NO’s role in tumorigenesis (Kim and Thomas, 2022; Ramírez-Patiño et al., 2022; Sahebnasagh et al., 2022).

Three isoforms of nitric oxide synthase (NOS), inducible NOS (iNOS), endothelial NOS (eNOS), and neuronal NOS (nNOS), have been detected in a variety of human cancers (Kim and Thomas, 2022; Ramírez-Patiño et al., 2022; Sahebnasagh et al., 2022). Among these, iNOS has been the most extensively studied in the context of tumor biology due to its capacity to produce sustained and high-output NO. Aberrant iNOS expression has been observed in colorectal, breast, and lung cancers, as well as in melanoma (Kim and Thomas, 2022; Ramírez-Patiño et al., 2022). Elevated iNOS expression has been detected in over 60% of advanced melanoma tumors and correlates with poorer patient outcomes (Ekmekcioglu et al., 2006). In contrast, studies in breast and colorectal cancers have reported an association between high iNOS expression and lower tumor grade or increased apoptosis (Alemu et al., 2025; Lin et al., 2022; Wang et al., 2020). These opposing findings have given rise to divergent therapeutic strategies targeting the iNOS/NO axis. On one hand, NOS inhibitors such as L-NAME and curcumin have been evaluated as antitumor agents but have shown limited clinical efficacy (Sharma et al., 2004; Kim and Thomas, 2022). On the other hand, NO-donating compounds such as JS-K and NO-ASA, designed to release cytotoxic concentrations of NO in tumor cells, have shown potential as therapeutic agents and are undergoing clinical evaluation (Gao and Williams, 2012; Maciag et al., 2013; Kim and Thomas, 2022). The coexistence of both pro- and anti-tumorigenic data highlights the need to better define the functional role of iNOS in specific tumor contexts and to validate reliable model systems for its study.

Conflicting evidence also surrounds the expression of iNOS in melanoma cells *in vitro*. Several studies have reported constitutive iNOS expression in A375 melanoma cells and in A375-derived xenograft tumors, as well as in primary patient-derived melanoma tissues (Ekmekcioglu et al., 2006; Uffort et al., 2009; Sikora et al., 2010; Godoy et al., 2012; Lopez-Rivera et al., 2014). However, other studies have failed to detect iNOS or NO production in A375 cells under comparable conditions (Chin and Deen, 2010). Given the well-documented capability of iNOS to produce high concentrations of NO in response to inflammatory stimuli, it remains unclear whether tumor cells that express iNOS can sustain such NO production without triggering apoptosis. This discrepancy raises questions about the biological relevance of iNOS expression in tumor cells and underscores the importance of validating iNOS expression *in vitro* using appropriate model systems.

Macrophages activated by inflammatory cytokines and bacterial components, such as lipopolysaccharide (LPS), interferon-gamma (IFN-γ), and interleukin-1β (IL-1β), have traditionally served as models for studying iNOS induction (Kashfi et al., 2021). However, these primary immune cells present limitations due to their complex handling and the lack of oncogenic signaling pathways that characterize tumor cells. Thus, a human carcinoma-based *in vitro* model capable of robust and inducible iNOS expression would offer a valuable platform for investigating NO signaling in a tumor-relevant context.

Interestingly, human iNOS was originally cloned from the colorectal adenocarcinoma cell line DLD-1 (Sherman et al., 1993), suggesting that this cell line may possess the molecular machinery necessary for iNOS induction. In the present study, we establish DLD-1 cells as an experimentally tractable *in vitro* model for inducible iNOS expression. We describe a method for inducing iNOS in these cells and provide systematic protocols for quantifying iNOS protein and mRNA, along with key downstream indicators of NO signaling, including nitrosative stress and nitrotyrosine-modified proteins. This model serves not only as a reliable positive control for validating iNOS expression across various systems but also offers a mechanistic tool for resolving conflicting findings in the literature and improving our understanding of NO’s diverse roles in cancer biology.

## 2 Materials and methods

### 2.1 Antibodies and reagents

Oligonucleotide primers for reverse transcription polymerase chain reaction (RT-PCR) were synthesized by Sigma-Aldrich (St. Louis, MO). Gradient polyacrylamide gels (4%–15%) for Western blotting were obtained from Bio-Rad Laboratories (Hercules, CA). Primary antibodies specific to human iNOS (sc-651) and murine iNOS (sc-650) were procured from Santa Cruz Biotechnology (Santa Cruz, CA), and an additional monoclonal antibody against human iNOS (clone 1E8-B8) was obtained from R&D Systems (Minneapolis, MN). An anti-nitrotyrosine antibody was purchased from Abcam (ab125106, Cambridge, MA).

For gene silencing experiments, Lipofectamine RNAiMAX transfection reagent was obtained from Life Technologies (Grand Island, NY). A non-targeting SMARTpool siRNA control and one set of ON-TARGETplus Human NOS2 siRNA (set of 4) were purchased from Dharmacon (Catalog number: LQ-009240-00-0005, Horizon Discovery, Lafayette, CO); an additional independent set of iNOS-targeting siRNAs, recognizing a different mRNA region, was purchased from Invitrogen (Catalog # 4392420, Assay ID: s9619 and s9620, Carlsbad, CA). Chemical inhibitors used in this study included S-methylisothiourea sulfate (SMT) and L-NG-nitroarginine methyl ester (L-NAME), both obtained from Calbiochem (San Diego, CA). Recombinant human cytokines, interleukin-1β (IL-1β, #A42508), and interferon-gamma (IFN-γ, # 300-02-500UG), and lipopolysaccharide (LPS, #00-4976-93) were purchased from eBioscience (San Diego, CA).

### 2.2 Cell culture

Human colorectal adenocarcinoma DLD-1 cells and human melanoma A375 cells were obtained from the American Type Culture Collection (ATCC, Manassas, VA) in 2013. Cell line authentication was performed via short tandem repeat (STR) DNA profiling using the AmpF/STR Identifiler PCR Amplification Kit (Applied Biosystems, Foster City, CA; cat. no. 4322288). STR analysis was conducted by the Characterized Cell Line Core Facility at The University of Texas MD Anderson Cancer Center.

Cells were cultured in Dulbecco’s Modified Eagle Medium (DMEM) supplemented with 5% fetal bovine serum (FBS), 100 μg/mL L-glutamine, 100 U/mL penicillin, and 100 U/mL streptomycin (Invitrogen). All cell lines were maintained at 37°C in a humidified atmosphere containing 5% CO_2_.

### 2.3 Induction of iNOS expression in DLD-1 cells

To evaluate iNOS expression, DLD-1 cells were seeded in 6-well culture plates at a density of 5 × 10^5^ cells per well and allowed to adhere overnight under standard conditions. The following day, cells were treated with a cytokine cocktail containing recombinant human interferon-gamma (IFN-γ, 20 ng/mL), interleukin-1β (IL-1β, 20 ng/mL), and lipopolysaccharide (LPS, 500 ng/mL) for various time intervals ranging from 4 to 24 h. These conditions were optimized to mimic pro-inflammatory stimulation known to induce iNOS expression. For comparative analyses, the same cytokine treatment regimen was applied to A375 melanoma cells under identical culture conditions.

### 2.4 Western blotting

Following treatment, cells were lysed in a buffer composed of 50 mM Tris-HCl (pH 7.9), 150 mM NaCl, 1% Nonidet P-40 (NP-40), 1 mM EDTA, 10% glycerol, and 1 mM sodium orthovanadate, supplemented with a protease inhibitor cocktail (Roche Diagnostics, Indianapolis, IN). Protein concentrations were determined using a standard Bradford assay. Equal amounts of total protein were resolved via SDS-PAGE using 4%–15% gradient polyacrylamide gels and transferred onto Hybond ECL nitrocellulose membranes (GE Healthcare Biosciences, Piscataway, NJ). Membranes were blocked in 5% nonfat dry milk diluted in phosphate-buffered saline (PBS) and incubated with primary antibodies specific to iNOS or nitrotyrosine, followed by appropriate horseradish peroxidase–conjugated secondary antibodies. Detection was performed using an enhanced chemiluminescence (ECL) reagent (GE Healthcare Biosciences), and protein bands were visualized using autoradiography or digital imaging systems.

### 2.5 Reverse transcription polymerase chain reaction (RT-PCR)

Total RNA was isolated from DLD-1 and A375 cells using the NucleoSpin RNA II extraction kit (Macherey-Nagel, Bethlehem, PA) according to the manufacturer’s protocol. First-strand cDNA was synthesized using 500 ng of total RNA and the GeneAmp RNA PCR kit (Applied Biosystems, Foster City, CA). A 2 μL aliquot of the resulting cDNA was used for each 25 μL PCR reaction.

Human iNOS mRNA was amplified using specific primer sets targeting different regions of the iNOS transcript, while β-actin was used as an internal loading control. Primer sequences are detailed in the Supplementary Information. PCR conditions included an initial denaturation step at 95°C for 5 min, followed by 30 amplification cycles consisting of denaturation at 95°C for 40 s, annealing at 55.5°C for 30 s, and extension at 72°C for 60 s. A final elongation step was performed at 72°C for 10 min. Amplified products (20 μL) were resolved on 1.5% agarose gels and visualized by ethidium bromide staining under UV illumination.

### 2.6 siRNA-mediated knockdown of iNOS

To evaluate the specificity of iNOS detection, DLD-1 and A375 cells were plated at a density of 2 × 10^5^ cells per well in 6-well plates and incubated overnight in 2 mL of complete DMEM at 37°C with 5% CO_2_. The following day, cells were transfected with 20 nM of human iNOS-specific small interfering RNA (siRNA) using 3.2 µL of Lipofectamine RNAiMAX (Invitrogen, Carlsbad, CA) per well, according to the manufacturer’s protocol. For protein band validation in Western blot assays, A375 cells were co-transfected with 20 nM siRNA targeting glyceraldehyde 3-phosphate dehydrogenase (GAPDH) and 20 nM human iNOS siRNA. Negative control groups included cells transfected with 20 nM non-targeting control siRNA or mock-transfected with Lipofectamine alone. Cells were harvested 24 h post-transfection for protein and mRNA analysis.

### 2.7 Quantification of nitrate/nitrite as indicators of NO production

Total NO production was estimated by measuring the accumulation of nitrate and nitrite, stable end products of NO metabolism, in cell culture supernatants. Two analytical approaches were employed for cross-validation. First, the Total Nitric Oxide and Nitrate/Nitrite Parameter Assay Kit (R&D Systems) was used according to the manufacturer’s protocol to colorimetrically detect nitrite/nitrate concentrations. Second, high-resolution detection was performed using the ENO-30 NOx Analyzer (Eicom, San Diego, CA), a dedicated HPLC-based system employing diazo coupling chemistry for selective quantification of nitrate and nitrite (Bryan and Grisham, 2007). For this analysis, equal volumes of culture supernatant and 100% methanol were mixed, vortexed, and centrifuged at 10,000 × g for 10 min. The resulting supernatant was injected into the analyzer via an AS-700 autosampler. Two distinct chromatographic peaks, representing nitrite and nitrate (converted to nitrite), were detected spectrophotometrically at 540 nm. Concentrations were quantified against standard curves generated from sodium nitrite and sodium nitrate standards.

### 2.8 Detection of intracellular reactive nitrogen species

The intracellular accumulation of reactive nitrogen species (RNS), including nitric oxide and its derivatives, was visualized in DLD-1 cells using the reactive oxygen species (ROS)/RNS Detection Kit (Enzo Life Sciences, Plymouth Meeting, PA). Following treatment, both stimulated and unstimulated cells were stained per the manufacturer’s protocol. A nitric oxide–specific fluorescent probe produced red fluorescence upon reaction with intracellular NO. Fluorescence signals were captured using a Nikon Eclipse TE 2000U inverted fluorescence microscope, and both red (NO/RNS) and green (control) fluorescence images were acquired to assess differential intracellular RNS levels.

### 2.9 Immunohistochemical detection of iNOS and nitrotyrosine

Immunohistochemistry (IHC) was performed as previously described (Ekmekcioglu et al., 2006) to detect protein expression of iNOS and nitrotyrosine in cell pellets or fixed specimens. Primary antibodies against human iNOS (sc-651) were used at dilutions of 1:50 and 1:200. Nitrotyrosine staining was performed using a 1:200 dilution of the corresponding antibody. Signal development was achieved using standard chromogenic detection methods, and slides were counterstained with hematoxylin prior to microscopic evaluation.

### 2.10 Statistical analysis

Quantitative data were analyzed using the two-tailed Wilcoxon rank-sum test after confirming normality to assess statistical differences in nitrate/nitrite levels among experimental groups. A p-value <0.05 was considered statistically significant. All statistical analyses were conducted using GraphPad Prism or equivalent statistical software.

## 3 Results

### 3.1 Inducible expression of human iNOS protein in DLD-1 cells

Given that the human iNOS cDNA was originally cloned from the DLD-1 colorectal adenocarcinoma cell line (Sherman et al., 1993), we investigated whether DLD-1 cells could serve as a robust model for studying iNOS expression and regulation. To this end, DLD-1 cells were stimulated with a combination of inflammatory mediators, LPS, IL-1β, and IFN-γ, and subjected to time-course analysis of protein expression.

**FIGURE 1:**
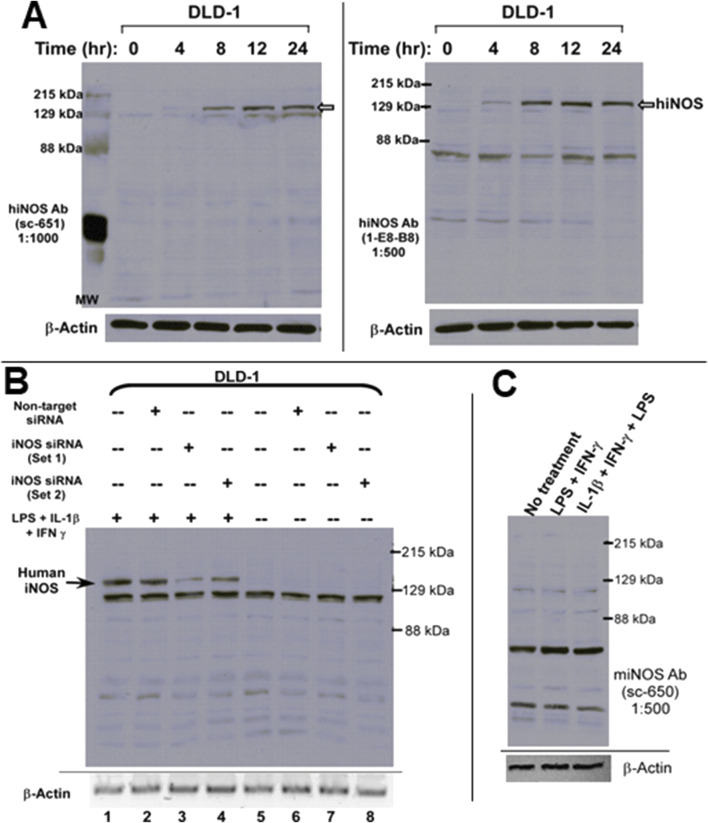
Inducible expression of human iNOS protein in DLD-1 colorectal carcinoma cells following inflammatory stimulation. **(A)** Time-course analysis of human iNOS (hiNOS) protein expression in DLD-1 cells after stimulation with a cytokine mixture containing LPS (500 ng/mL), IL-1ẞ (20 ng/mL), and IFN-y (20 ng/mL). Western blots of total cell lysates (40 μg/lane) were probed with two independent anti-hiNOS antibodies: sc-651 (left panel) and 1E8-B8 (right panel). A prominent band of ∼130 kDa emerged as early as 4 hours post-stimulation, with progressive accumulation observed up to 24 h, consistent with inducible hiNOS expression. **(B)** Validation of iNOS protein identity using RNA interference. DLD-1 cells were transfected with two independent siRNA sets targeting distinct regions of the hiNOS mRNA. Western blotting revealed that both siRNA sets effectively suppressed the 130-kDa iNOS band in cytokine- stimulated cells without affecting other non-specific bands detected by the hiNOS antibody, demonstrating the specificity of the observed signal. **(C)** Species specificity of INOS antibody detection. Western blot analysis using an antibody specific to mouse iNOS (sc-650) failed to detect any corresponding band in cytokine-stimulated DLD-1 cells, confirming that the iNOS signal observed in **(A,B)** represents human iNOS and not cross-reactivity with murine proteins. B-actin served as a loading control throughout all panels.

Western blot analysis revealed the induction of a distinct protein band corresponding to ∼130 kDa, consistent with the expected molecular mass of human iNOS, emerging at 4 h post-stimulation and reaching maximal expression by 24 h (Figure 1A). This expression pattern was confirmed using two independent human iNOS-specific antibodies (sc-651 and 1E8-B8), affirming both the inducibility and temporal dynamics of iNOS in this model. Importantly, the specificity of this signal was validated by RNA interference using two distinct siRNA sets targeting different regions of the human iNOS transcript. Both siRNA sets effectively knocked down iNOS expression in stimulated DLD-1 cells, while non-targeting siRNA had no effect (Figure 1B). This knockdown confirmed the identity of the ∼130 kDa band as human iNOS.

To further ensure species specificity, we probed the DLD-1 lysates with a mouse-specific iNOS antibody (sc-650), which failed to detect any corresponding band in human cells (Figure 1C), supporting the selective detection of human iNOS in this system. Notably, none of the Western blot analyses revealed dimeric forms of human iNOS under the experimental conditions tested, suggesting that the enzyme predominantly exists in its monomeric state in this model.

Despite the effective detection of iNOS with two commercial antibodies, we observed that both antibodies also cross-reacted with several unrelated proteins that were not depleted by human iNOS-targeting siRNAs (Figure 1B). This underscores the potential for non-specific signals around the 130 kDa region, which could lead to misinterpretation in systems lacking appropriate human iNOS-positive controls. Therefore, the use of validated controls, such as stimulated DLD-1 cells, is critical when assessing iNOS expression by immunoblotting.

**FIGURE 2:**
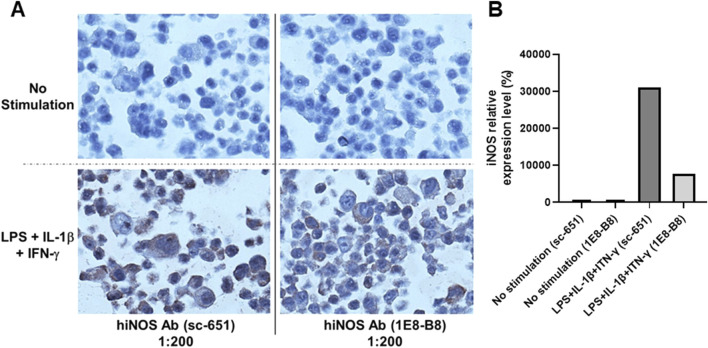
Immunohistochemical detection of human iNOS in DLD-1 cells following cytokine stimulation. **(A)** Representative IHC staining of DLD-1 cells with anti-hiNOS antibodies (sc-651 and 1E8-B8, 1:200 dilution) following treatment with or without LPS, IL-1ẞ, and IFN-y. In unstimulated cells (top panels), no detectable iNOS staining was observed, whereas stimulated cells (bottom panels) exhibited strong cytoplasmic iNOS immunoreactivity, confirming inducible protein expression consistent with the immunoblotting data shown in Figure 1. **(B)** Quantitative analysis of iNOS immunostaining intensity from **(A)** expressed as fold-change (%) relative to the unstimulated control. These findings validate the DLD-1 model as a reliable system for studying stimulus-dependent iNOS induction in human carcinoma cells.

The presence of iNOS protein in DLD-1 cells following cytokine stimulation was further corroborated by immunohistochemical analysis (Figures 2A,B). Robust cytoplasmic staining for iNOS was evident in stimulated cells, while unstimulated controls displayed no detectable signal, consistent with the absence of iNOS protein under basal conditions.

Collectively, these results establish DLD-1 cells as a reliable and responsive *in vitro* model for studying inducible human iNOS expression. The inducibility, specificity, and reproducibility of iNOS protein detection in this model system provide a valuable platform for investigating nitric oxide signaling and resolving inconsistencies reported in other cancer cell lines.

### 3.2 Inducible expression of full-length iNOS mRNA in DLD-1 cells

Human iNOS is encoded by a gene spanning 27 exons, which together encode a 1153-amino-acid enzyme critical for NO production in immune and tumor biology (Sherman et al., 1993). To determine whether DLD-1 colorectal carcinoma cells can be used as a model for studying full-length human iNOS transcription, we designed a panel of eight primer sets that amplify overlapping segments of the entire iNOS coding sequence. These primer sets target regions of the human iNOS transcript reported in the NCBI database (RefSeq NM_000625.4) and were selected to also detect possible splicing variants, as previously described in literature (Fiddler, 1977; Sherman et al., 1993; Eissa et al., 1996; Pautz et al., 2010) (primer sequences provided in Supplementary Information).

**FIGURE 3:**
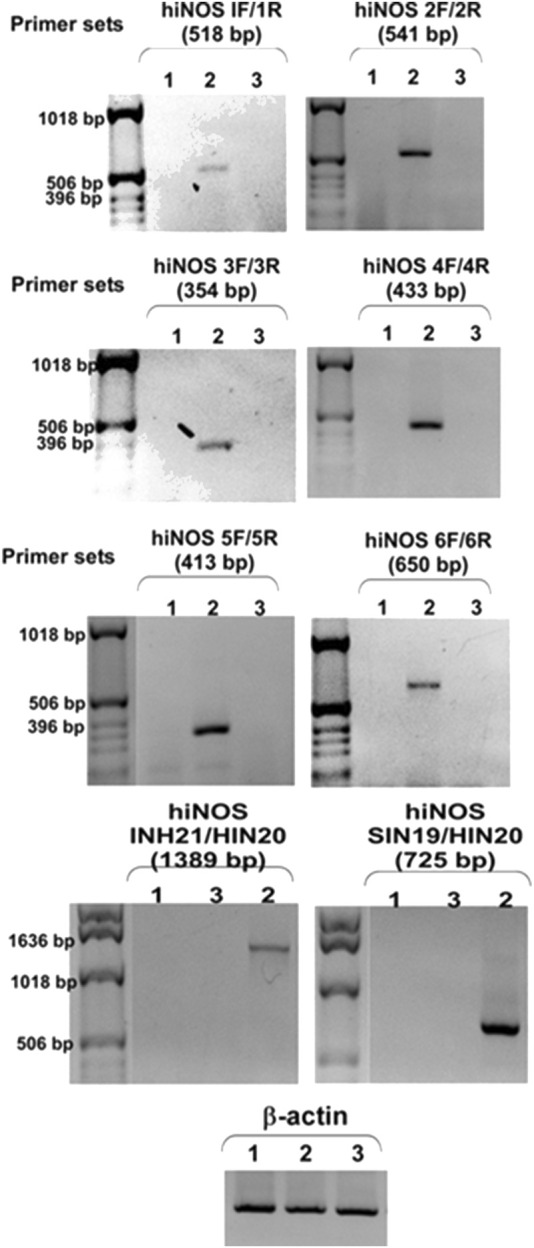
Inducible expression of full-length hiNOS mRNA in DLD-1 cells confirmed by RT-PCR. Total RNA was extracted from DLD-1 cells cultured under three conditions: unstimulated (lane 1), stimulated with IL-1ẞ (20 ng/mL). IFN-y (20 ng/mL), and LPS (500 ng/mL) for 24 h (lane 2), and stimulated DLD-1 cells transfected with iNOS-specific siRNA (lane 3). RNA was subjected to reverse transcription followed by PCR using six primer sets designed to amplify different regions of the full-length human iNOS coding sequence (NM_000625.4). Amplification products of expected sizes were obtained exclusively from stimulated DLD-1 cells (lane 2), confirming inducible and full-length transcription of hiNOS. No specific bands were observed in RNA from unstimulated or iNOS- silenced cells (lanes 1 and 3), indicating absence of basal expression and validating siRNA-mediated knockdown. B-actin served as a control for RNA quality and input normalization.

RT-PCR analysis revealed that six out of the eight primer sets produced amplification products of the expected sizes (ranging from 354 bp to 1389 bp) when cDNA from cytokine-stimulated DLD-1 cells was used as the template (lane 2, Figure 3). The amplified products were validated by sequencing and confirmed to match the canonical human iNOS transcript sequence. No splicing variants or truncations were detected under our stimulation conditions, supporting prior reports that human iNOS mRNA exhibits limited alternative splicing following inflammatory induction (Fiddler, 1977).

Importantly, no amplification was observed in cDNA samples from unstimulated DLD-1 cells (lane 1, Figure 3), indicating that human iNOS mRNA is not expressed under basal conditions. Furthermore, cDNA from DLD-1 cells treated with iNOS-targeting siRNA after stimulation also failed to produce amplification products (lane 3, Figure 3), supporting the specificity of the detected transcripts as human iNOS mRNA. The β-actin transcript, used as an internal loading control, was consistently detected in all samples, confirming RNA integrity and even input across conditions.

Collectively, these results demonstrate that DLD-1 cells can robustly express full-length human iNOS mRNA in response to stimulation with IL-1β, IFN-γ, and LPS. The DLD-1 cell line therefore represents a robust and inducible human carcinoma model system for studying the transcriptional regulation of iNOS and its downstream functional pathways.

### 3.3 iNOS induction in DLD-1 cells increases NO production and nitrosative stress, which are attenuated by iNOS inhibition

**FIGURE 4:**
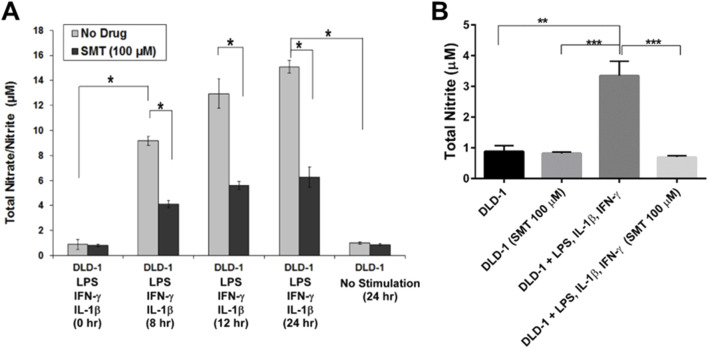
Induction of iNOS in DLD-1 cells leads to increased production of nitrate/nitrite, which is attenuated by iNOS inhibition. **(A)** Quantification of total nitrate/nitrite levels in DLD-1 cell culture supernatants following stimulation with LPS (500 ng/mL), IL-1ẞ (20 ng/mL), and IFN-y (20 ng/mL) for 0, 8, 12, or 24 h. The light gray bars represent cells stimulated in the absence of inhibitor, while the black bars represent cells co- treated with the selective iNOS inhibitor S-methylisothiourea sulfate (SMT; 100 μM). Nitrate/nitrite concentrations were measured using the Griess assay. Data are presented as mean ± SEM from three independent experiments performed in triplicate. **(B)** Nitrite levels were independently validated using a NOx analyzer (HPLC-based method). Cells were treated under the indicated conditions for 24 h, and supernatants were analyzed for nitrite accumulation. SMT significantly suppressed nitrite production in stimulated DLD-1 cells. Data are expressed as mean ± SEM from three independent experiments (n=3). *p < 0.05, **p < 0.01, ***p < 0.001.

To evaluate the functional relevance of iNOS expression in DLD-1 cells, we assessed NO production by measuring total nitrate and nitrite concentrations in cell culture supernatants, commonly used as stable proxies for NO generation. Using the Griess assay, we observed that DLD-1 cells stimulated with a pro-inflammatory cytokine cocktail (IFN-γ, IL-1β, and LPS) produced significantly higher levels of nitrate/nitrite in a time-dependent manner, with maximal accumulation at 24 h post-stimulation (Figure 4A). In contrast, unstimulated cells exhibited only basal levels of nitrate/nitrite. Co-treatment with S-methylisothiourea sulfate (SMT; 100 μM), a selective iNOS inhibitor, led to a marked reduction in nitrate/nitrite levels across all time points (p = 0.0116), implicating iNOS as the primary contributor to NO production in this model (Figure 4A).

To further validate these findings, we employed a high-performance liquid chromatography-based NOx analyzer to quantify nitrite levels. Consistently, DLD-1 cells subjected to cytokine stimulation exhibited a substantial increase in extracellular nitrite, which was significantly diminished upon SMT treatment (p = 0.0238) (Figure 4B). These results confirm that cytokine stimulation induces robust iNOS-dependent NO production in DLD-1 cells, which can be pharmacologically suppressed by iNOS inhibition.

**FIGURE 5:**
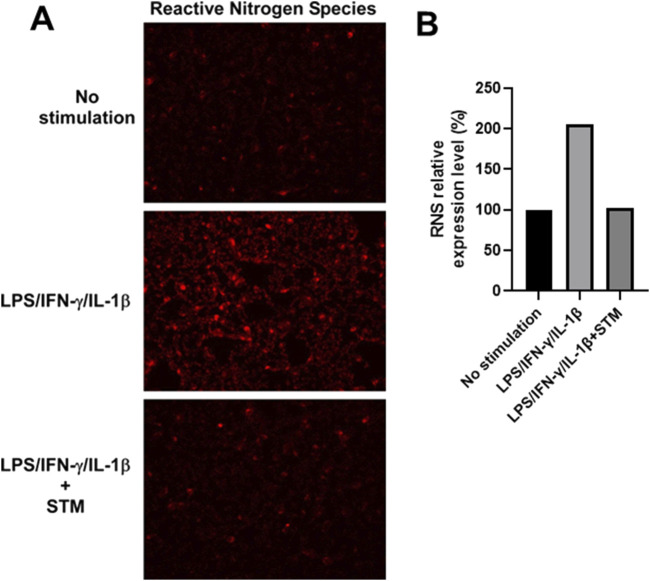
Increased production of reactive nitrogen species (RNS) in DLD-1 cells upon inflammatory stimulation is suppressed by iNOS inhibition. **(A)** Fluorescence microscopy analysis of total RNS levels in DLD-1 cells under the indicated conditions using the ROS/RNS Detection Kit. Red fluorescence corresponds to intracellular nitric oxide and RNS accumulation. **(B)** Quantitative analysis of red fluorescence intensity from **(A)** shown as fold-change (%) relative to unstimulated controls. DLD-1 cells stimulated with LPS, IL-1ẞ, and IFN-y showed a substantial increase in red fluorescence, indicating elevated NO production. Co-treatment with SMT (100 μM) markedly reduced RNS-associated fluorescence intensity, supporting the specificity of iNOS-derived NO in driving RNS generation.

Next, we assessed the intracellular accumulation of RNS using a fluorescence-based detection method. Stimulation of DLD-1 cells with IFN-γ, IL-1β, and LPS led to a pronounced increase in red fluorescence, indicative of elevated RNS levels (Figures 5A,B). This fluorescence signal was markedly reduced in cells co-treated with SMT, demonstrating that the observed RNS accumulation is primarily dependent on iNOS-derived NO.

**FIGURE 6:**
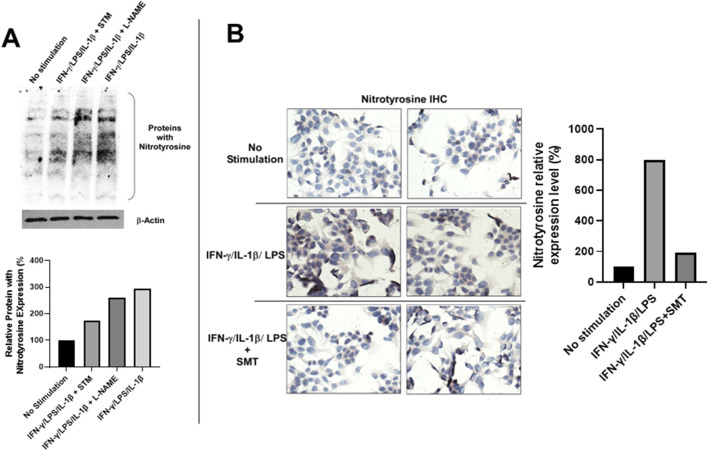
iNOS-derived nitric oxide in DLD-1 cells promotes tyrosine nitration, which is attenuated by pharmacological inhibition. **(A)** Western blot analysis of total protein tyrosine nitration in DLD-1 cells under the indicated treatment conditions. Increased nitrotyrosine levels were observed following 24- hour stimulation with LPS, IL-1ẞ, and IFN-y. This effect was significantly reduced by SMT (100 μM) and moderately reduced by the pan-NOS inhibitor L- NAME (100 μM). B-actin served as a loading control. Quantification of nitrotyrosine levels relative to ẞ-actin is presented below the blot. **(B)** IHC staining of nitrotyrosine-modified proteins in DLD-1 cells. Strong cytoplasmic staining was observed in cells stimulated with LPS, IL-1ẞ, and IFN-y, while staining was markedly reduced in cells co-treated with SMT (100 μM). No staining was detected in unstimulated cells. Anti-nitrotyrosine antibody was used at a dilution of 1:200. Quantitative analysis of nitrotyrosine immunostaining intensity of **(B)** expressed as fold-change (%) relative to the unstimulated control.

To determine whether iNOS-derived NO contributes to downstream nitrosative stress, we evaluated protein tyrosine nitration by detecting nitrotyrosine, a stable biomarker of peroxynitrite (ONOO^−^)-mediated protein modification (Campolo et al., 2020). Western blot analysis revealed a significant increase in nitrotyrosine-modified proteins in stimulated DLD-1 cells relative to unstimulated controls, and this upregulation was partially abrogated by SMT treatment (Figure 6A). Treatment with the pan-NOS inhibitor L-NAME (100 μM) also reduced nitrotyrosine levels, albeit to a lesser extent. These findings were corroborated by IHC staining, which demonstrated intense nitrotyrosine immunoreactivity in stimulated DLD-1 cells and marked attenuation upon SMT treatment (Figure 6B). Quantitative densitometry normalized to β-actin confirmed the differential expression of nitrotyrosine across experimental groups (Figure 6A).

Collectively, these data demonstrate that inflammatory stimulation of DLD-1 cells induces functionally active iNOS, resulting in elevated NO production, accumulation of reactive nitrogen species, and protein nitration. Importantly, these effects can be selectively mitigated by iNOS-targeted pharmacologic inhibition. These results establish DLD-1 as a robust inducible human carcinoma model to investigate NO biology and to evaluate iNOS-targeted therapeutic strategies in inflammation-driven cancers.

## 4 Discussion

**FIGURE 7:**
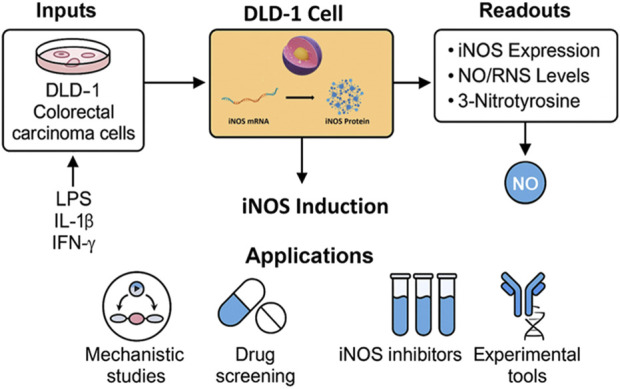
Graphical summary of the DLD-1-based in vitro platform for inducible iNOS expression and NO signaling analysis. The workflow illustrates cytokine stimulation, iNOS induction, and key downstream readouts, with applications in drug screening, mechanistic studies, and tool validation.

In this study, we systematically characterized iNOS expression at both the mRNA and protein levels in the human colorectal carcinoma cell line DLD-1 following stimulation with LPS, IL-1β, and IFN-γ. We further evaluated the functional consequences of iNOS induction by measuring NO production and associated nitrosative stress markers. Our results establish that DLD-1 cells, upon inflammatory stimulation, robustly express full-length, enzymatically active human iNOS, and generate biologically significant levels of NO and downstream products. This inducible model offers a reproducible, easily maintained, and well-controlled *in vitro* system for studying iNOS/NO signaling and testing NO-modulatory agents in the context of human carcinoma. To visually consolidate our findings, we present a graphical summary (Figure 7) illustrating the experimental workflow and potential applications of the DLD-1-based platform for studying iNOS regulation and nitrosative signaling in cancer cells.

In addition to DLD-1 cells, variable levels of inducible nitric oxide synthase (iNOS) expression have been documented across several other human colorectal cancer (CRC) cell lines, including HT-29, HCT116, and SW480 (Ambs et al., 1998; Kobelt et al., 2020; Du et al., 2023). Notably, HCT116 and HT-29 cells exhibit minimal or undetectable iNOS expression under basal conditions and demonstrate only limited iNOS induction following stimulation with pro-inflammatory cytokines such as IFN-γ and TNF-α (Ambs et al., 1998; Kobelt et al., 2020; Du et al., 2023). These observations highlight the intrinsic heterogeneity in iNOS responsiveness among CRC subtypes, which may stem from underlying genetic and epigenetic differences that modulate key upstream regulatory pathways, particularly NF-κB and STAT1 signaling. Such disparities underscore the importance of cellular context in iNOS regulation. While the DLD-1 model provides a reliable and highly inducible system for studying NO signaling, broader comparative analyses across multiple CRC cell lines are essential to assess the generalizability of the findings and to fully understand the spectrum of iNOS regulation within colorectal cancer biology.

A limitation of the current study is the use of a two-dimensional (2D) monoculture system, which lacks the complexity of the TME. *In vivo*, iNOS expression is regulated not only by tumor-intrinsic signaling but also by dynamic interactions with infiltrating immune cells, stromal components, hypoxia, and cytokine gradients, all of which are absent in conventional *in vitro* models. The immunosuppressive or pro-inflammatory nature of the TME, in particular, plays a pivotal role in modulating NO production and its downstream effects on tumor progression or immune evasion (Kashfi et al., 2021; Navasardyan and Bonavida, 2021; Li et al., 2025). Therefore, while the DLD-1 system offers a controlled platform for mechanistic interrogation and screening of NO-modulating agents, its physiological relevance may be improved by integrating 3D culture systems, co-culture with immune cells, or *in vivo* models that better recapitulate the spatial and cellular heterogeneity of colorectal tumors. The DLD-1 cell model offers a valuable platform for future studies aimed at validating iNOS protein identity, post-translational modifications, and abundance using mass spectrometry–based proteomics. This approach would provide orthogonal confirmation beyond antibody-based methods and enable higher-resolution characterization of iNOS-related signaling. Given the known limitations of antibody specificity and cross-reactivity, particularly in the context of closely related NOS isoforms, such proteomic validation is critical to ensure accurate detection and quantification in cancer models.

The dual nature of NO in tumor biology is well-documented, with low concentrations promoting cell survival and angiogenesis, and high concentrations triggering apoptosis and immune activation (Kim and Thomas, 2022; Ramírez-Patiño et al., 2022; Sahebnasagh et al., 2022). The complexity of NO’s biological effects is further compounded by the context-dependent activity of iNOS. Although iNOS has been detected in a variety of cancers—including colorectal, breast, lung, and melanoma—its prognostic significance remains controversial (Ekmekcioglu et al., 2006; Ramírez-Patiño et al., 2022; Sahebnasagh et al., 2022). For example, while high iNOS levels in advanced melanoma correlate with poor survival (Ekmekcioglu et al., 2006), elevated iNOS expression in breast and colorectal tumors has been linked to favorable prognostic features and increased apoptosis (Wang et al., 2020; Lin et al., 2022; Alemu et al., 2025). These inconsistencies underscore the need for well-validated experimental systems to dissect the roles of iNOS in cancer progression.

One of the key questions in tumor immunobiology is whether constitutively expressed iNOS in cancer cells produces functionally active NO at levels comparable to those generated by inducible iNOS in activated macrophages (Uffort et al., 2009; Sikora et al., 2010; Godoy et al., 2012; Lopez-Rivera et al., 2014). While no definitive evidence indicates that tumor-derived iNOS is catalytically inactive, the possibility exists that tumor cells have evolved adaptive mechanisms to buffer or exploit high-output NO synthesis for tumor-promoting processes. Our findings support the use of stimulated DLD-1 cells as a robust iNOS-positive control to investigate such mechanisms.

A major factor contributing to discrepancies in the literature is the variable accuracy and specificity of iNOS detection methods. Western blotting and IHC are commonly used to evaluate protein expression in tumor tissues, yet both approaches are susceptible to technical variability and antibody cross-reactivity. In our analysis, several commercial human iNOS antibodies exhibited nonspecific binding to unrelated proteins, complicating the interpretation of Western blot and IHC results. Importantly, only a subset of antibodies reliably detected the ∼130 kDa iNOS protein in stimulated DLD-1 cells, and this specificity was validated using siRNA-mediated knockdown. These results highlight the necessity of including appropriate positive and negative controls, such as stimulated and unstimulated DLD-1 cells, to ensure accurate interpretation of IHC or Western blot data.

While IHC offers spatial localization of protein expression, quantitative polymerase chain reaction (qPCR) provides superior sensitivity and specificity for evaluating transcript levels. A recent qPCR study reported low iNOS mRNA expression in 95% of primary melanoma samples, contrasting with earlier IHC-based findings that suggested high iNOS expression in metastatic melanoma (Ekmekcioglu et al., 2006; Dabbeche-Bouricha et al., 2016). It remains to be determined whether this discrepancy reflects genuine differences between primary and metastatic tumors, or methodological artifacts related to antibody specificity and tissue processing. Given its robustness and quantitative output, qPCR should be prioritized over IHC for assessing iNOS expression, especially in settings where antibody performance is uncertain.

Accurate and direct detection of intracellular NO remains a substantial technical challenge in cancer research. NO is a highly reactive, short-lived free radical gas that diffuses rapidly and undergoes immediate interaction with intracellular targets, making its real-time quantification within cells particularly difficult. Conventional approaches to assess NO activity rely heavily on indirect markers, including the accumulation of its stable oxidation products, nitrite (NO_2_^−^) and nitrate (NO_3_^−^), or detection of post-translational modifications such as protein tyrosine nitration (e.g., nitrotyrosine) and S-nitrosylation of cysteine residues (Bryan and Grisham, 2007; Bradley and Steinert, 2016; Prolo et al., 2024). These downstream indicators, however, do not always correlate linearly with the instantaneous intracellular NO concentrations, particularly under dynamic redox conditions present in tumor cells.

*In vitro* studies commonly utilize exogenous NO donors, such as S-nitroso-N-acetylpenicillamine (SNAP), diethylenetriamine-NONOate (DETA-NONOate), or sodium nitroprusside (SNP), often at micromolar (µM) concentrations, to mimic NO exposure and evaluate its impact on cancer cell proliferation, apoptosis, or immune signaling (Ridnour et al., 2006; Goloshvili et al., 2019; Ding et al., 2021). Nonetheless, it is widely recognized that the actual intracellular concentrations of NO achieved under such conditions are likely to be significantly lower than the applied donor concentrations. This discrepancy arises due to the rapid diffusion and neutralization of NO by cellular antioxidants, including glutathione, as well as interactions with metalloproteins and ROS in the culture medium or within cells (Zhang and Hogg, 2004; Thomas et al., 2008; Wang et al., 2024). Moreover, the buffering capacity of the cellular redox system and the heterogeneity of NO bioavailability in different subcellular compartments further complicate accurate measurement and interpretation. Consequently, developing more selective, high-resolution tools for the real-time detection of intracellular NO in cancer cells remains a critical unmet need for advancing our understanding of NO-mediated signaling and its dual role in tumor progression and immune regulation.

Our study also validates DLD-1 cells as a reliable and responsive *in vitro* model for mechanistic studies of inflammation-induced NO signaling in colorectal cancer. Importantly, the DLD-1 inducible system offers a tractable platform to dissect the upstream regulatory signaling pathways that govern iNOS expression, particularly nuclear factor kappa B (NF-κB) and signal transducer and activator of transcription 1 (STAT1). These transcription factors are key mediators of cytokine- and pathogen-associated molecular pattern (PAMP)-driven responses and are known to directly regulate iNOS gene transcription via binding to consensus elements within the *NOS2* promoter [PMID: 7508926, 12048217, 28778215]. Future investigations using this model can incorporate specific pharmacologic inhibitors, RNA interference, or CRISPR-based gene editing to delineate the relative contributions of NF-κB and STAT1, as well as their crosstalk, in modulating iNOS induction and downstream metabolic changes.

Elevated iNOS protein expression has been reported in approximately 30%–45% of human metastatic CRC cases and is significantly associated with poor patient survival (p < 0.01) (Ropponen et al., 2000; Zafirellis et al., 2010). Multiple studies have demonstrated that nitric oxide (NO), produced via iNOS, can promote tumor cell proliferation, resistance to apoptosis, and contribute to an immunosuppressive TME in CRC (Ropponen et al., 2000; Zafirellis et al., 2010; Mandal, 2018). In addition, pharmacological inhibition of NOS has been shown to attenuate angiogenesis and tumor progression in preclinical CRC models (Gao et al., 2019). These findings underscore the pathological role of iNOS/NO-driven nitrosative stress in CRC biology. Notably, beyond the selective iNOS inhibitor SMT and the pan-NOS inhibitor L-NAME used in our study, at least 14 other NOS inhibitors with greater isoform selectivity, particularly toward iNOS, have been evaluated in various *in vitro* and *in vivo* CRC models (Wang et al., 2020). Despite promising preclinical results, none of these compounds have progressed into clinical trials for CRC treatment (Wang et al., 2020), highlighting a significant translational gap. Future studies leveraging our DLD-1 model could support mechanistic comparisons of these inhibitors and help identify candidates with favorable pharmacodynamic profiles. Broader efforts to validate NOS inhibition strategies in clinically relevant settings are urgently needed to advance their potential as therapeutic agents in CRC.

Taken together, our results position the DLD-1 cell line as a validated and practical model for studying inducible iNOS expression and NO-related signaling in human epithelial cancer cells. The availability of this system enables rigorous evaluation of NO-modulating compounds, exploration of iNOS-dependent mechanisms in tumor immunology, and benchmarking of experimental tools such as antibodies and primers. By providing a consistent and inducible iNOS expression platform, this model may help reconcile conflicting findings across different tumor types and experimental conditions, and ultimately clarify the functional role of iNOS in cancer biology.

## Data Availability

The original contributions presented in the study are included in the article/Supplementary Material, further inquiries can be directed to the corresponding author.
