# Supplementary material for: Development of a human colorectal carcinoma cell-based platform for studying inducible nitric oxide synthase expression and nitric oxide signaling dynamics
Source: Front Mol Biosci. 2025 Jul 17;12:1637230. doi: 10.3389/fmolb.2025.1637230 (PMC12310501; doi:10.3389/fmolb.2025.1637230)
Supplement: Supplementary file 1 [file Presentation1.ppt]

## Slide 1
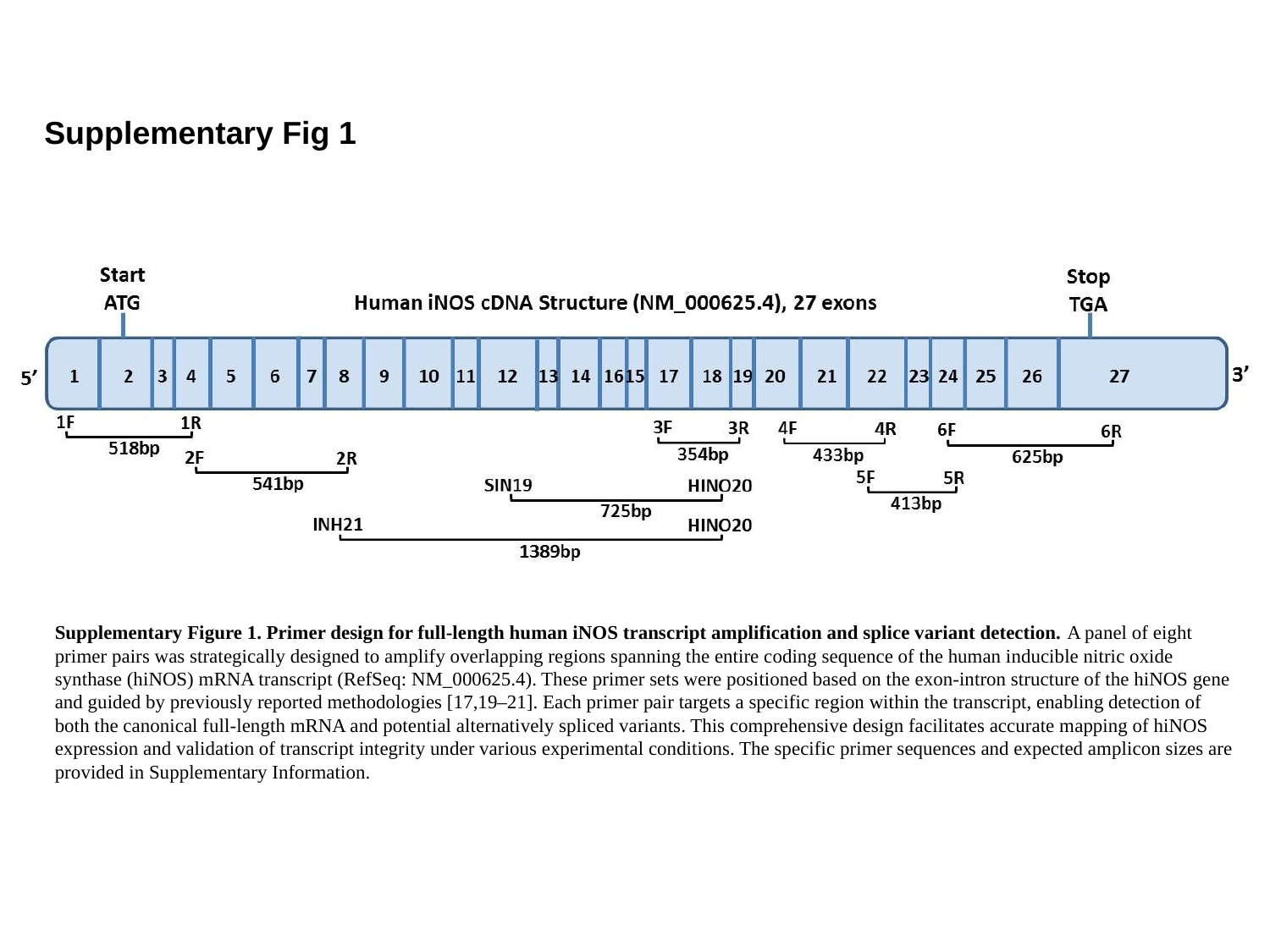

Supplementary Fig 1
Supplementary Figure 1. Primer design for full-length human iNOS transcript amplification and splice variant detection. A panel of eight primer pairs was strategically designed to amplify overlapping regions spanning the entire coding sequence of the human inducible nitric oxide synthase (hiNOS) mRNA transcript (RefSeq: NM_000625.4). These primer sets were positioned based on the exon-intron structure of the hiNOS gene and guided by previously reported methodologies [17,19–21]. Each primer pair targets a specific region within the transcript, enabling detection of both the canonical full-length mRNA and potential alternatively spliced variants. This comprehensive design facilitates accurate mapping of hiNOS expression and validation of transcript integrity under various experimental conditions. The specific primer sequences and expected amplicon sizes are provided in Supplementary Information.

## Slide 2
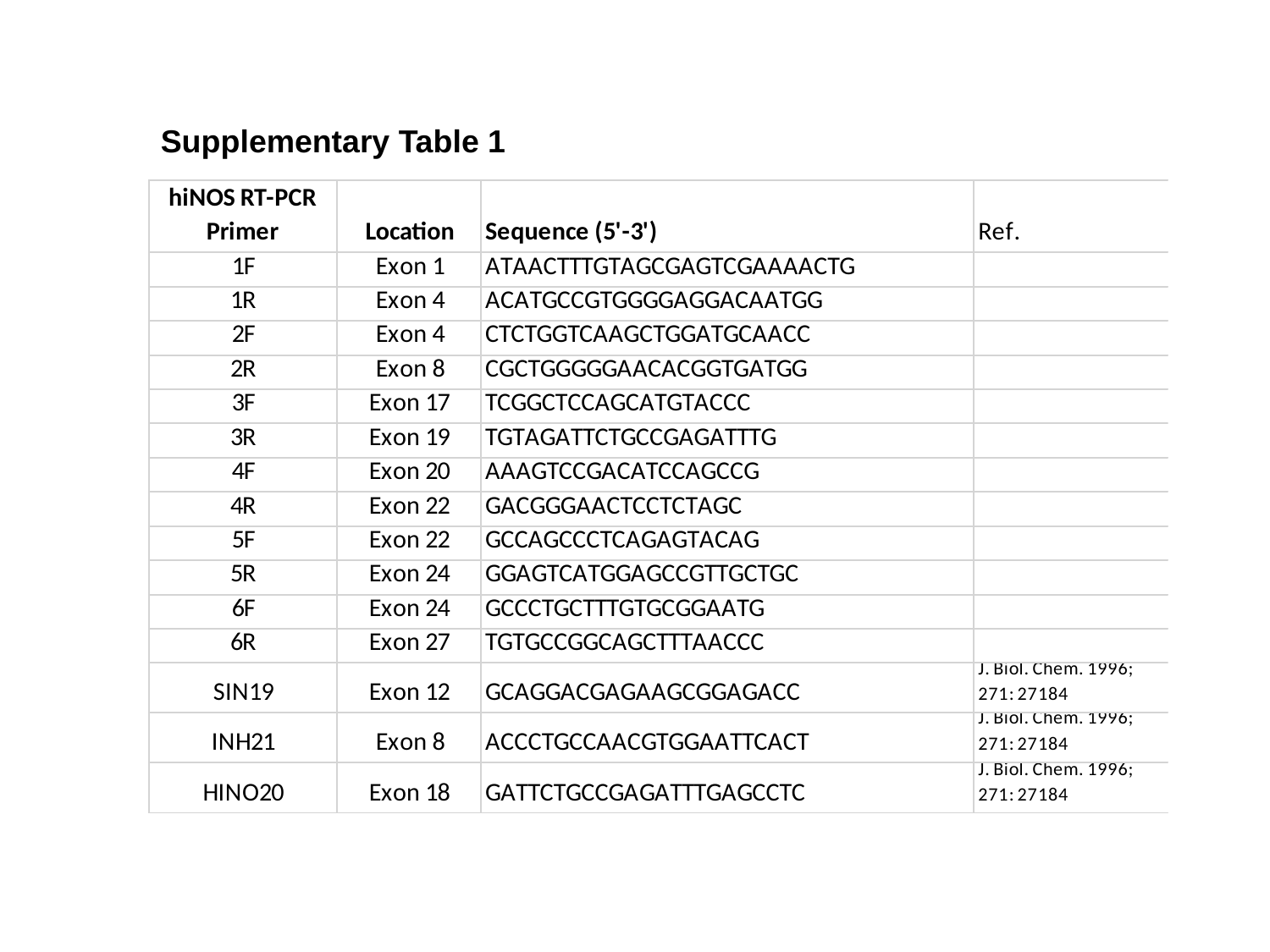

Supplementary Table 1
